# Risk factors for bipolar hemiarthroplasty dislocation: a retrospective cohort study

**DOI:** 10.1007/s00590-026-04853-4

**Published:** 2026-07-06

**Authors:** Andreas Baranowski, Céline Stahn, Erik Wegner, Philipp Schippers, Victoria Buschmann, Eren Demir, Austin Harper, Ulrike Ritz, Erol Gercek, Philipp Drees, Tobias Nowak

**Affiliations:** 1 University Medical Center of the Johannes Gutenberg University, Mainz, Germany; 2 Department of Trauma Surgery, Orthopedics and Reconstructive Surgery, ANregiomed Hospital, Ansbach, Germany; 3 Department of Anaesthesiology, Intensive Care Medicine, Pain Therapy and Palliative Medicine, Diakonie Hospital, Bad Kreuznach, Germany; 4 SGU’s School of Medicine, St. George’s University, Saint George’s, Grenada

**Keywords:** Bipolar hemiarthroplasty, Femoral‑neck fractures, Risk factors, Dislocation, Morphological parameters

## Abstract

**Purpose:**

Bipolar hemiarthroplasty (BHA) is widely used for displaced femoral-neck fractures; however postoperative dislocation remains a serious complication. Reported risk factors vary considerably. This study investigated the clinical, surgical and radiographic parameters associated with dislocation following BHA.

**Methods:**

We conducted a retrospective single‑center cohort study of 434 patients who underwent BHA for femoral‑neck fractures at a level‑I trauma center between 1 January 2016 and 31 December 2020. Demographics, comorbidities, surgical approach (timing and duration), and postoperative radiographs were analyzed. Patients with and without dislocation were compared using univariate analyses. Receiver operating characteristic (ROC) analysis and logistic regression were performed to assess predictive value. In a nested case-control subanalysis, each dislocation case was matched with five age and sex matched controls for detailed comparison analysis.

**Results:**

The cohort included 165 men and 269 women (mean age 81.9 years); dislocation occurred in 2.5% of patients (*n* = 11). Age, sex, dementia, Parkinson’s disease, time to surgery and operative duration were not associated with dislocation. Operations performed outside daytime hours were more frequent among dislocated cases (45% vs. 20%, *p* = 0.016). Dislocated hips had a smaller center–edge angle (CEA), larger femoral neck-shaft angle (FNSA), reduced contralateral offset and higher bipolar head extrusion index. A CEA ≤ 25° predicted dislocation with high specificity but low sensitivity. Logistic regression suggested nighttime surgery as an additional potential predictor.

**Conclusion:**

Pre‑operative evaluation of CEA and consideration of operative timing may help identify patients at increased risk of dislocation following BHA. Prospective randomized trials are required to confirm these associations, clarify causality, and develop preventive strategies.

## Introduction

Hip fractures represent a major healthcare burden in industrialized nations, and their incidence is still increasing due to demographic changes [1, 2]. Displaced femoral-neck fractures account for a large proportion of these injuries, with approximately 160.000 cases occurring every year in Germany [3]. Early mobilization and weight-bearing are particularly important for functional rehabilitation in frail or multimorbid patients. For elderly patients with limited life expectancy and low functional demands, bipolar hemiarthroplasty (BHA) has become the gold-standard surgical treatment [4]. Unlike osteosynthesis, BHA offers immediate stability and avoids complications associated with prolonged immobilization. On the other hand, arthroplasty introduces its own spectrum of complications, with dislocation among the most serious, occurring in approximately 4.5% (0.76 to 2.2%) [5, 6]. Disloction typically occurs early postoperatively and necessitates closed or open reduction, thus prolonging hospitalization and increasing patient morbidity [7]. A substantial body of literature in trauma surgery and fracture epidemiology has examined risk factors for BHA dislocation, yet findings remain inconsistent. Suggested patient‑related predictors of instability include advanced age, female sex, dementia or Parkinson’s disease, and delayed surgery [8–10]. Other studies have implicated surgical factors such as operative approach [11], implant design, operative timing [12], and radiographic alignment [13–15]. Anatomical and biomechanical investigations have highlighted the role of acetabular coverage [16], femoral offset and femoral neck–shaft angle (FNSA) in maintaining stability, while large cohort studies have demonstrated the importance of timely surgery [12] and comorbidity burden [10]. Epidemiological studies have provided a long list of clinical risk factors, while emphasizing the added value of fall prevention programs [17–19]. Thresholds of CEA vary; some authors recommend cut‑offs ≤ 45° [20, 21], while others have suggested lower thresholds [13, 22], reflecting the challenges of generalizing results across populations.

In view of these uncertainties, we conducted a comprehensive retrospective analysis of all patients who underwent BHA for femoral‑neck fractures at a high‑volume trauma center over a five‑year period. We hypothesized that both patient‑related and surgical factors influence dislocation risk. The aims of this study were threefold: (i) to describe the demographic and clinical characteristics of the cohort; (ii) to identify associations between dislocation and demographic, clinical and surgical parameters; and (iii) to assess whether radiographic morphology (CEA, FNSA, femoral offset and extrusion indices) predicts dislocation. A nested case–control subanalysis was undertaken to investigate radiographic parameters in greater detail.

## Materials and methods

### Study design and setting

This is a retrospective cohort study carried out at the [*anonymized*], which is a tertiary referral hospital that provides comprehensive care to a predominantly geriatric population. All consecutive patients with femoral‑neck fractures treated with BHA at the center from January 1, 2016 to December 31, 2020 were included.

### Patient identification and inclusion criteria

Patients were identified from the hospital’s electronic information system using ICD‑10-GM (International Statistical Classification of Diseases and Related Health Problems, 10th revision, German Modification) codes S72.00–S72.08 for medial femoral‑neck fractures and the operative procedure code for BHA. This search yielded 434 patients. No patients were excluded based on age, sex or comorbidities. All included patients received a cemented or cementless bipolar prosthesis inserted via an anterolateral Watson‑Jones or lateral transgluteal Bauer approach under general or spinal anesthesia.

### Data collection

Demographic data (age, sex), medical comorbidities (dementia and Parkinson’s disease), operative timing (date and time) and operative duration (skin incision to closure) were retrieved from electronic medical records. To ensure there was complete ascertainment of all dislocation events, institutional records were reviewed, all available imaging studies were evaluated, and digital procedure codes for both closed and open reductions were verified. Time to surgery was defined as the interval from the initial diagnostic imaging to first skin incision. Surgical procedures were categorized according to start time as either daytime (8:00am-5:00PM) or nighttime (outside these hours). Those commencing outside of these hours were classified as nighttime surgeries. Postoperative complications were recorded, with dislocation defined as any event requiring closed or open reduction.

### Surgical protocol

All operations were carried out in line with a standardized institutional protocol. Patients received preoperative antibiotic prophylaxis and underwent either a Watson‑Jones (anterolateral) or Bauer (transgluteal) approach, depending on surgeon preference. In 432 patients, cemented stems were implanted, while two patients received cementless stems (Taperloc^®^ Hip Femoral Stem, Zimmer Biomet, Warsaw, United States). A bipolar head (RingLoc^®^, Zimmer Biomet, Warsaw, United States) was used in all cases. Standardized anterior–posterior pelvic radiographs and lateral hip radiographs were taken pre‑ and post‑operatively. Postoperative management included early mobilization with full weight‑bearing, standard postoperative wound care and thromboprophylaxis with low‑molecular‑weight heparin for 28–35 days, or resumption of pre‑existing anticoagulation after completion of wound healing.

### Morphological measurements

**Fig. 1:**
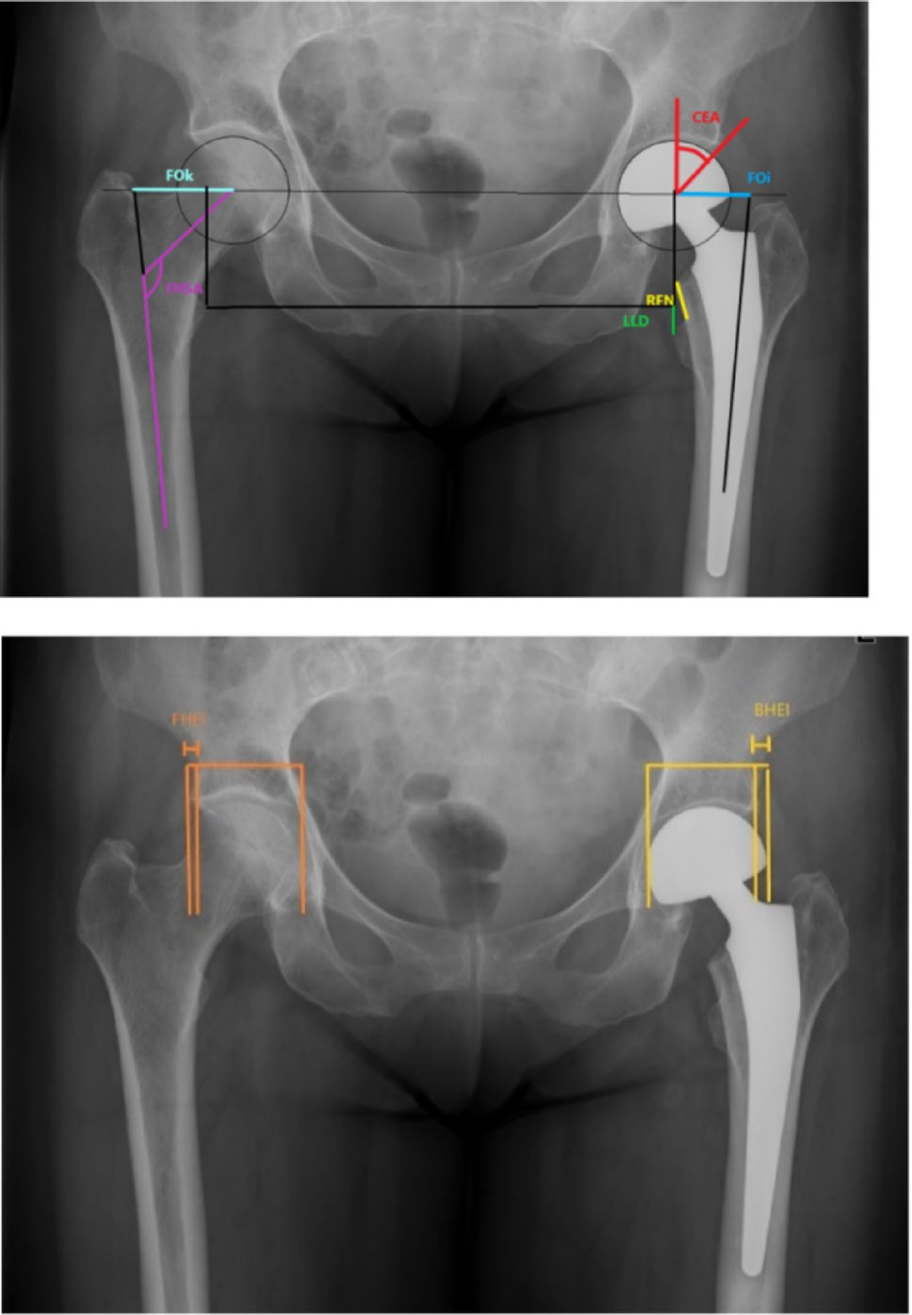
Measurement of radiographic parameters. Abbreviations: BHEI = bipolar head extrusion index, FHEI = femoral head extrusion index

Radiographic measurements were performed on calibrated images by a single observer, using Sectra ^®^ software, to reduce inter‑rater variability (Fig. 1a, b). Intra-observer reliability was not formally assessed in this study. The CEA was defined as the angle between a vertical line through the center of the femoral head and a line connecting the head center to the lateral acetabular rim. The FNSA described the angle between the femoral neck axis and femoral shaft axis on the contralateral side. Femoral offset (FO) was measured as the perpendicular distance from the center of the femoral head to the intramedullary femoral axis, separately for the ipsilateral (FOi) and contralateral (FOc) sides. Residual femoral‑neck length represented the remaining neck length after implantation. Leg‑length discrepancy (LLD) was calculated as the vertical difference between the lesser trochanters on an anterior–posterior pelvis radiograph; positive values indicated a longer operated limb. Bipolar head extrusion index (BHEI) and femoral head extrusion index (FHEI) quantified the proportion of the prosthetic or native head uncovered by the acetabulum.

Figure 1a. Measurement of radiographic parameters. Abbreviations: CEA = center edge angle, FNSA = femoral neck-shaft angle, FOi = femoral offset ipsilateral, FOk = femoral offset contralateral, RFN = residual femoral-neck length, LLD = leg-length discrepancy.

### Statistical analysis

Statistical analyses were performed using R (version 4.0.2) or GraphPad Prism (version 9.5.0). All figures were generated with the R package “ggplot2”. The distribution of continuous variables was tested for normality using the Kolmogorov–Smirnov test, and data are presented as mean ± standard deviation (SD) or median with interquartile range (IQR), depending on the normality of the distribution. Categorical variables were presented as frequencies and percentages. Comparisons of continuous variables between patients with and without dislocation were performed by unpaired t‑tests, Mann‑Whitney U tests, or Fisher’s Exact test, while chi‑square tests assessed the association between categorical variables. The predictive value of CEA cut‑off values for dislocation was evaluated using ROC analysis. All variables with a p value ≤ 0.2 on univariate analysis were entered into a multivariable logistic regression model with statistical significance set at *p* < 0.05. To enable more detailed morphometric comparisons with greater statistical power, a nested case–control subanalysis was carried out. Each dislocation case (*n* = 11) was matched to five controls (*n* = 55) based on age and sex. The same univariate tests as in the entire cohort were used to compare morphological parameters.

## Results

### Patient characteristics

Of the 434 patients who underwent BHA, 165 were men and 269 women (Table 1). The median age was 81.9 years (interquartile range 78–87) in the control group and 79.6 years (interquartile range 75–88) in the dislocation group, with no statistically significant difference between the groups (*p* = 0.36). Dementia was present in 180 patients (41.8%), and Parkinson’s disease in 38 patients (9.0%); neither comorbidity was associated with dislocation. Eleven patients (2.5%) experienced prosthetic dislocation during follow‑up.

**Table 1:** Demographic patient characteristics

Variable / Groups	Control (*n* = 423)	Dislocation (*n* = 11)	*p*-Value
Age (years / IQR)	81.9 (18–87)	79.6 (75–88)	0.36
Dementia (n / % of group)	177 (41.8%)	3 (27.3%)	0.33
Parkinson’s disease (n / % of group)	38 (9.0%)	0 (0%)	0.30
Sex			0.17
Female (n / % of group)	260 (61.5%)	9 (81.8%)	
Male (n / % of group)	163 (38.5%)	2 (18.2%)	

Data are presented either as absolute numbers with the corresponding percentage relative to the respective group or as median with interquartile range. Abbreviations: IQR = interquartile range.

### Temporal and operative factors

Most dislocations occurred within the first ten days after surgery. The period between surgery and dislocation ranged from one to 36 days, with eight of eleven dislocations occuring within the first postoperative week. The median time from diagnosis to surgery was 24.4 h (interquartile range 17.4–42) in controls and 23.1 h (interquartile range 11.3–25) among dislocation cases, showing no statistically signifcant difference (*p* = 0.37). Median operative duration was 70 min (interquartile range 55–85) in controls and 76 min (interquartile range 58–87) in dislocation cases, again not different from each other. However, operations performed outside daytime hours were markedly overrepresented among patients with dislocation: 45% of patients with dislocations underwent surgery during the night shift, whereas only 20% of patients in the control were operated on at night (OR = 4.57, 95% CI 1.36–15.32, *p* = 0.016).

### Radiographic parameters in the full cohort

**Fig. 2:**
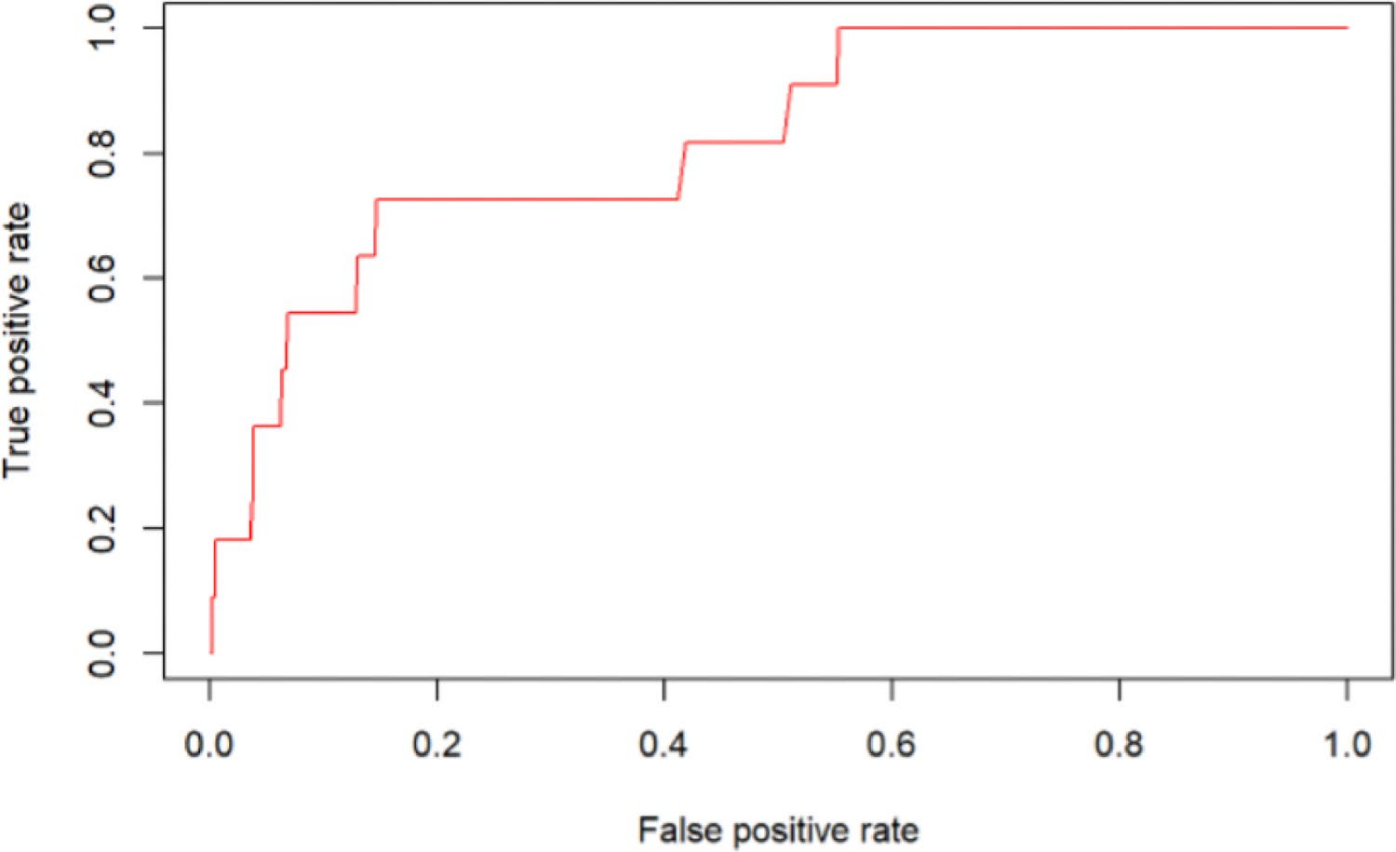
ROC curve of the relation of true (sensitivity) and false (specifity) positive rate in the prediction of dislocations

**Fig. 3:**
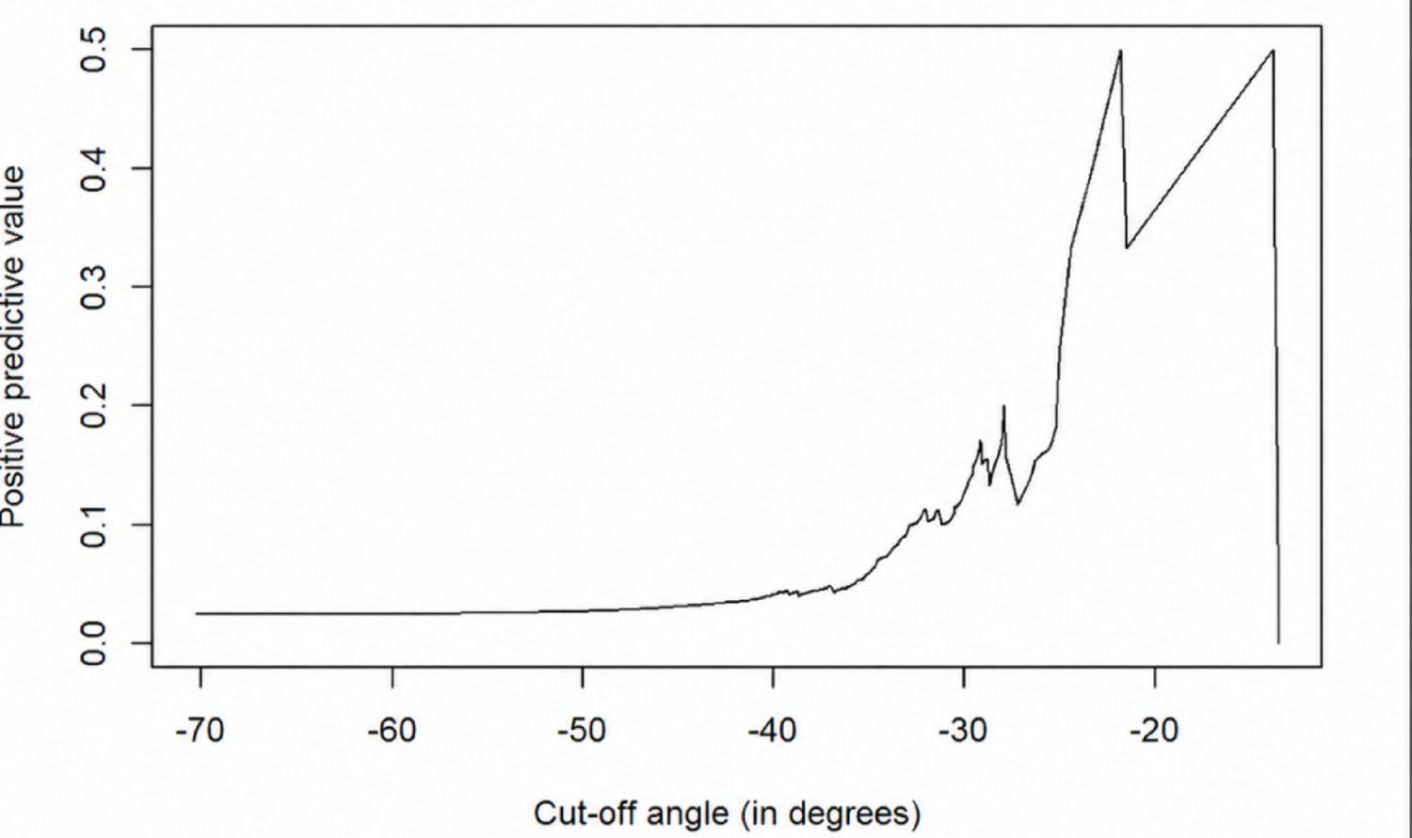
Positive predictive value (PPV) of the CEA cut-off angles (in degrees). The low event rate inherently limits the positive predictive values achievable, even when using the optimal cut-off. For example, a CE angle of < 25° can be used to achieve a PPV of approximately 30%

The mean CEA was 38.8° in the whole cohort. In controls the median CEA was 38.7° (interquartile range 34.6–43.3) compared with 29.2° (interquartile range 27.8–37.0) in the dislocation group (*p* = 0.0001). Application of the threshold of ≤ 45° identified all dislocations but yielded many false positives (Figs. 2 and 3), whereas a lower cut‑off of ≤ 25° maximized specificity but at the cost of sensitivity (Figs. 2 and 3). At this threshold, the model achieved:

- Sensitivity: 1.00.
- Specificity: 0.16.
- Positive Predictive Value (PPV): 0.03.
- Negative Predictive Value (NPV): 1.00.
- Accuracy: 0.18.
- Balanced accuracy: 0.58.

This cut-off was intentionally chosen to maximize sensitivity, ensuring that no positive cases were missed. However, this comes at the expense of a low specificity and a high false-positive rate.

### Logistic regression

Stepwise logistic regression retained CEA and operative timing as independent predictors of dislocation. Our model suggested that nighttime operations conferred additional risk beyond that associated with acetabular undercoverage. Other variables, including age, sex, dementia, Parkinson’s disease, time to surgery, and operative duration, were not retained.

### Nested case–control subanalysis

This nested subanalysis of 66 patients comprised 11 cases of dislocation and 55 matched controls (Table 2). Regarding the surgical approach, 29 patients (52.7%) in the control group, compared with 4 patients (36.4%) in the dislocation group, underwent an anterolateral approach. The lateral transgluteal approach was used in 26 control patients (47.3%) and in 7 patients with dislocation (63.6%). No significant difference in dislocation rate was observed with respect to surgical approach (*p* = 0.32). The mean CEA in this subcohort was 38.5 ± 6.5° in controls versus 29.8 ± 7.5° in dislocated cases (*p* = 0.0002). Patients with dislocated hips had a larger femoral neck–shaft angle (141.3 ± 6.4° vs. 132.4 ± 5.2°, *p* < 0.0001). Ipsilateral femoral offset (FOi) did not differ between groups (31.6 ± 6.0 mm vs. 28.3 ± 9.1 mm, *p* = 0.14). Whereas contralateral offset (FOc) was lower in dislocation cases (median 32.6 mm vs. 38.6 mm, *p* = 0.0054). The residual femoral-neck length and leg-length discrepancy showed no significant differences between the groups. In contrast, the bipolar head extrusion index (BHEI) was higher in dislocated cases (18.5 ± 7.5 versus 12.1 ± 4.7, *p* = 0.0004), while the femoral head extrusion index (FHEI) did not differ between groups.

**Table 2:** Nested case–control subanalysis of matched BHA groups

Variable / Groups	Control (*n* = 55)	Dislocation (*n* = 11)	*p*-Value
Surgical approach (%)			0.32
Anterolateral	29 (52.7%)	4 (36.4%)	
Lateral transgluteal	26 (47.3%)	7 (63.6%)	
Radiographic parameters			
CEA in degree (SD)	38.5 ± 6.5	29.8 ± 7.5	< 0.001***
FNSA in degree (SD)	132.4 ± 5.2	141.3 ± 6.4	< 0.0001****
FOi in mm (SD)	31.6 ± 6.0	28.3 ± 9.1	0.14
FOc in mm (IQR)	38.6 (32.3–41.2)	32.6 (25.5–35.2)	< 0.01**
BHEI in mm (SD)	12.1 ± 4.7	18.5 ± 7.5	0.001***
FHEI in mm (IQR)	9.5 (5.4–13.6)	10.8 (6.5–15.6)	0.44
LLD in mm (SD)	5.0 ± 5.9	3.7 ± 3.9	0.44

Data are reported either as mean ± standard deviation or as median with interquartile range. Abbreviations: BHA = bipolar hemiarthroplasty, IQR = interquartile range, SD = Standard deviation, CEA = center edge angle, FNSA = femoral neck-shaft angle, FOi = femoral offset ipsilateral, FOc = femoral offset contralateral, BHEI = bipolar head extrusion index, FHEI = femoral head extrusion index, LLD = leg length discrepancy; **p < 0.01, ***p < 0.001 ****p < 0.0001

## Discussion

This single‑center retrospective study investigated risk factors for dislocation following BHA in 434 consecutive patients treated for femoral‑neck fractures over a five‑year period. The overall dislocation rate was low (2.5%), consistent with reported ranges of about 4.5% [5, 6, 23–25]. We found that patient demographics (age and sex), comorbid dementia and Parkinson’s disease, time to surgery, surgical approach and operative duration were not associated with the risk of dislocation, in line with several previous studies [26, 27]. However, operations performed outside daytime hours were overrepresented among the cases of dislocation and logistic regression identified nighttime surgery as an independent predictor. Given the limited number of dislocations, the findings should be interpreted as exploratory. The association between nighttime surgery and dislocation is not necessarily causal and nighttime surgery may act as a proxy for contextual variables such as staffing, experience, or case urgency. The influence of operative timing on outcomes remains controversial; some studies suggest that delay to surgery prolongs hospital stay and may increase mortality [12, 15]. Our finding that nighttime surgery is associated with dislocation underscores the need to further investigate whether scheduling surgery during daytime hours might reduce complications. Nevertheless, current guidelines recommend surgery within 24 h, and delaying surgery solely for scheduling reasons may not be justified.

Our data suggest that acetabular under-coverage, reflected by a smaller CEA, is associated with an increased risk of postoperative dislocation. The CEA was about 9° lower in dislocated hips compared to controls, echoing previous literature identifying a shallow acetabulum as a major risk factor [8, 13, 15, 20, 21]. The ROC analysis demonstrates that no single cut‑off value achieves both high sensitivity and high specificity, reflecting the heterogeneity of patient populations and measurement techniques. In the nested subanalysis, additional morphologic differences were observed: a larger femoral neck–shaft angle and higher bipolar head extrusion index were associated with dislocation, whereas ipsilateral offset, residual neck length and leg‑length discrepancy were not. A smaller contralateral offset was also correlated with dislocation; although its clinical relevance remains uncertain, similar observations have been recorded in a study of Pajarinen et al. [11].

The present study has several strengths. The uniformity of the implant design and consistent use of standard surgical approaches used for all operations minimized heterogeneity. The cohort represents real‑world practice as no patients were excluded because of age or comorbidities. Comprehensive electronic records enabled detailed clinical and radiographic data collection. Limitations include the retrospective design, under‑reporting of dislocation events occurring outside of our institution and the small absolute number of dislocations, reduced the power of multivariable analyses. Hence, the findings should not be interpreted as confirmatory and require validation in larger prospective cohorts. Radiographic measurements were obtained from standard anterior–posterior pelvis radiographs, which tend to underestimate femoral offset by up to 13% compared with computed tomography. A formal assessment of intra‑observer reliability was not performed, which represents a methodological limitation of this study. The nested case–control design increased efficiency but may be subject to residual confounding.

Our findings suggest practical implications. Preoperative pelvic radiographs should be examined for acetabular coverage; as patients with very small CEA may benefit from alternative implant options (e.g., tripolar prosthesis) which offer increased stability. Surgeons may also consider optimizing femoral offset intra‑operatively through implant choice to tension the abductors, though the clinical benefit remains unproven and must be weighed against limb‑length discrepancies. Scheduling surgery during daytime hours may be associated with a lower risk of dislocation, but prospective studies are needed to confirm this finding and to clarify whether operative timing reflects underlying factors such as surgeon experience, workload, or fatigue. Future research should address whether modifications in implant selection or surgical technique can reduce dislocation rates.

## Conclusion

The current study suggests that acetabular undercoverage and nighttime surgery may be associated with an increased risk of dislocation following bipolar hemiarthroplasty. Awareness of these risk factors can help clinicians refine surgical strategies. However, no single parameter can predict or prevent dislocation because it is multifactorial. Strong prospective data are required to guide clinical decision-making and optimize outcomes in this vulnerable patient population.

## Data Availability

The datasets generated during and/or analyzed during the current study are available from the corresponding author on reasonable request.

## Abbreviations

BHA: Bipolar hemiarthroplasty
BHEI: Bipolar head extrusion index
CEA: Center-edge angle („Wiberg angle“)
CI: Confidence interval
FHEI: Femoral head extrusion index
FNSA: Femoral neck-shaft angle
FOi: Femoral offset, ipsilateral
FOc: Femoral offset, contralateral
IQR: Interquartile range
LLD: Leg length discrepancy
OR: Odds ratio
ROC: Receiver operating characteristic
SD: Standard deviation
